# Developing a Health Game to Prepare Preschool Children for Anesthesia: Formative Study Using a Child-Centered Approach

**DOI:** 10.2196/31471

**Published:** 2022-01-20

**Authors:** Brynja Ingadottir, Elina Laitonen, Adalheidur Stefansdottir, Anna Olafia Sigurdardottir, Berglind Brynjolfsdottir, Heidi Parisod, Johanna Nyman, Karitas Gunnarsdottir, Katrín Jónsdóttir, Sanna Salanterä, Anni Pakarinen

**Affiliations:** 1 Faculty of Nursing School of Health Sciences University of Iceland Reykjavik Iceland; 2 Landspitali University Hospital Reykjavik Iceland; 3 Department of Nursing Science University of Turku Turku Finland; 4 City of Reykjavik Reykjavik Iceland; 5 Children's Hospital Landspitali University Hospital Reykjavik Iceland; 6 Nursing Research Foundation Helsinki Finland; 7 Turku University Hospital Turku Finland

**Keywords:** anesthesia, child-centred design, children, digital health, educational games, health games, hospital, patient education, serious games, surgery, user-centred design, video games

## Abstract

**Background:**

Every year, millions of children undergo medical procedures that require anesthesia. Fear and anxiety are common among young children undergoing such procedures and can interfere with the child’s recovery and well-being. Relaxation, distraction, and education are methods that can be used to prepare children and help them cope with fear and anxiety, and serious games may be a suitable medium for these purposes. User-centered design emphasizes the involvement of end users during the development and testing of products, but involving young, preschool children may be challenging.

**Objective:**

One objective of this study was to describe the development and usability of a computer-based educational health game intended for preschool children to prepare them for upcoming anesthesia. A further objective was to describe the lessons learned from using a child-centered approach with the young target group.

**Methods:**

A formative mixed methods child (user)-centered study design was used to develop and test the usability of the game. Preschool children (4-6 years old) informed the game design through playful workshops (n=26), and usability testing was conducted through game-playing and interviews (n=16). Data were collected in Iceland and Finland with video-recorded direct observation and interviews, as well as children’s drawings, and analyzed with content analysis and descriptive statistics.

**Results:**

The children shared their knowledge and ideas about hospitals, different emotions, and their preferences concerning game elements. Testing revealed the high usability of the game and provided important information that was used to modify the game before publishing and that will be used in its further development.

**Conclusions:**

Preschool children can inform game design through playful workshops about health-related subjects that they are not necessarily familiar with but that are relevant for them. The game’s usability was improved with the participation of the target group, and the game is now ready for clinical testing.

## Introduction

Young children worldwide undergo anesthesia in relation to various medical procedures, including surgery, dental procedures, physical examination, and medical imaging, and the uncertainty of the upcoming procedure may cause anxiety and distress. The reported prevalence of such anxiety is up to 50% in preschool children [1]. Children may experience fear because of the unfamiliar hospital equipment, environment, and staff, as well as pain, separation from parents and family, and being left alone [2,3]. In the short term, the child’s anxiety can cause difficulties in the induction of anesthesia and may precipitate perioperative complications, such as delirium and postoperative pain [4,5]. In the long term, behavioral changes, such as apathy, bedwetting, sleep disturbances, feeding difficulties, temper tantrums, and fear of medical personnel, are well known [6,7].

Among successful nonpharmacological interventions that can reduce procedure-related anxiety in children are education about the upcoming procedure and cognitive behavioral therapy [8]. Education about the upcoming procedure can take place in discussion with health care professionals and parents [9]. Discussions help children separate imagination from reality, build trust, reduce uncertainty, and increase children’s belief that they can cope with stressful situations [10]. Cognitive behavioral therapy, such as positive self-talk, relaxation, and deep-breathing exercises, have also been successful in reducing procedural distress and pain in children [11]. However, such techniques need to be taught and practiced before the procedure in order to be beneficial. Combined educational approaches, such as play therapy with information provided through video, modeling, puppet shows, or visits to the operating theater, have also been successful in reducing children’s anxiety [8,12-14]. The limitations of many such programs are poor accessibility and high costs, including human resources [15], but information technology, such as educational games, offers new and more feasible approaches to preparing children for medical procedures [8].

Games are a medium that can be useful for teaching and supporting coping skills and thus strengthening self-efficacy, health literacy, and knowledge in young children. Only a few health games have been developed to help prepare children for hospitalization and surgery. Promising results in improving children’s knowledge and tackling their anxiety have, for example, been achieved with the health games HospiAvontuur [16] and CliniPup [17]. A virtual reality approach, presenting the preoperative process with gamification, has also been effective in reducing anxiety in young children 5-8 years old [18].

The input of users is acknowledged as fundamental for success in designing technology. User-centered design addresses the whole user experience during the iterative process of design and development and includes multidisciplinary perspectives and skills [19]. It focuses on gaining an understanding of the user of the product and involves the user throughout the design and development phases [20].

Child-centered design derives from user-centered design and acknowledges the notion that it is desirable to incorporate children’s perspective, rights, and needs in the design process [21] in order to increase the usability and acceptability of the technology, and also that young children are able to participate and give their opinions if the design and involvement is presented in a child-oriented manner. Children can be involved as users, testers, informants, or design partners, depending on their participation at different stages of the design process [22]. Involving young, preschool children may be challenging [23] as their physical, cognitive, social, and emotional development and skills, such as motor skills or social skills, vary, both within the same age group as well as between different ages in general [21]. Abilities in abstract and logical thinking, and translating experiences into verbal statements, are not yet fully developed in young children [24], and they may have difficulties putting their feelings into words [23]. This means that when assessing their engagement during testing of a product, more than 1 evaluation method is required [23]. An observation of a set of behaviors, rather than their responses to questions, may be a better way to understand their true thoughts [24]. Therefore, children’s level of engagement during testing a product or participating in the design can be monitored or assessed with behavior such as frowns and yawns [25], fiddling, shrugs or ear-playing [26], vocalization (either positive or negative), concentration, smiling, or laughing [24]. When designing with children, using age-appropriate language, avoiding unfamiliar and abstract concepts, and paying attention to their existing attitudes, beliefs, previous experiences, and motives related to the health topic may all influence children’s understanding and their willingness to use the health information [24]. These can be explored during the user-centered design process.

An objective of this formative study was to describe the development and usability of a computer-based educational health game intended for preschool children to prepare them for upcoming anesthesia. A further objective was to describe the lessons learned from using a child-centered approach with the target group.

## Methods

### Study Team

A multidisciplinary team of researchers, pediatric and emergency nurses, a pediatric psychologist, a preschool teacher, and nurse anesthetists from Iceland and Finland initially identified the need for improved preparation of children for anesthesia and created the basic idea and concept of the game. In collaboration with a private game company and a composer, the team was expanded for the purpose of designing and implementing game techniques, graphics, and music.

The development and evaluation of the game followed a child-centered design, a process starting with the identification of a need and leading to a product that meets child-specific requirements [19,27]. The approach used included 2 steps: (1) specifying product requirements and producing design solutions through participatory design (workshops) and (2) evaluating the usability of the design by children through usability testing.

To specify the context of use, information was first collected on the current situation in clinical practice from both within the research group and with interviews with clinicians (1 anesthetist and 2 anesthetist nurses). In addition, the parents of 17 children were interviewed about their experiences of having and managing children undergoing anesthesia. Throughout the design process, the game was tested by children in the target group and simultaneously the interview frame used in the usability testing was created. The game was developed, tested, and published in 2019-2020.

### Development of the Game

#### Workshops

Preschool children were invited to inform the design process and to specify the product requirements through playful workshops after the initial idea of the game had been formed. The aim of the workshops was to explore the children’s general perceptions and knowledge about hospitals and emotions that were relevant to the game project. The workshops were planned with parallel protocols in both countries and first piloted with a comparable group of children. Minor adjustments were made to the setup and tasks after that.

Preschools in Iceland and Finland were contacted for collaboration purposes to assist with finding participants and to provide locations for the workshops. A purposive sampling method [28] was implemented, and those eligible for participation were children aged 4-6 years who could understand Icelandic/Finnish. The workshops were held in a preschool classroom and facilitated in Iceland by 1 preschool teacher and 2 nurses from the research group and in Finland by 1 nurse researcher. During the workshops, data were collected with direct observation (free text notes), interviews (open-ended questions with probing to deepen, develop, or clarify the interview answers), photos, and the children’s drawings. The researchers documented the age and gender of the children. All the workshop sessions were audio-video-recorded.

The workshops included 3 tasks: a board game, a poster walk, and drawing (Table 1).

**Table 1:** An overview of the workshops.

Task	Aim	Equipment	Children, n	Facilitator, n	Duration, minutes
Board game	Explore ideas about hospitals and emotions.	Dice, counters, board, bag with hospital equipment, 2 boxes with questions (on hospitals and emotions)	4-5	1	20-30
Poster walk	Explore preferences and emotions.	Printed posters presenting different types of characters, color palettes, and surroundings Posters used to address the subject of emotions	2	1	10
Drawing	Explore ideas about hospitals through drawings and conversations.	Paper and crayons	2-3	1	10

Each workshop had a group of 4-6 children who knew each other so that everyone would feel comfortable talking and communicating. The classroom was prepared before the children entered, with a big printed-out board game on the floor, and pictures with different color schemes and figures were hung on the walls. Sitting on the floor, after an introduction, the workshop facilitator explained the purpose of the workshop to the children and that it would be video-recorded and asked for the children’s consent for doing so. The children were asked whether they wanted to participate and told they could quit any time they liked.

#### Board Game

The board game was chosen as a medium to explore the children’s perceptions and knowledge about hospitals and their understanding of emotions related to the game project (ie, fear, excitement, pain, courage, relaxation, and anxiety). Playing the board game included throwing a pair of dice, moving a counter on a premarked surface, and, depending on the color of the surface, getting a different type of question to answer about (1) hospital and illness, (2) common hospital equipment, and (3) emotions. When playing the board game, the researchers tried to get into a fairytale way of thinking and talking to encourage the children’s creative thinking. The questions about hospital and illness were open ended to get the children to express their thoughts and ideas and included the following: *Why do people go to hospitals? What happens in a hospital? What people would you see, or meet in the hospital? What does a nurse and a doctor do? What is a medicine and why do you get it? What “things” (equipment) are in a hospital?* When the children got a question about hospital equipment, they were asked, *What do you think this is used for (demonstrating a stethoscope, a facemask, an intravenous cannula, a syringe, an anesthesia balloon with an attached mask, or a Band-Aid)?* The children were also asked about each of the 6 emotions relevant for the game, for example, *Sometimes we need to be brave; do you know what that means? Sometimes we are scared of something; what does that mean?* During the gameplay, the discussions were also directed to the children’s favorite foods and drinks (to use in the preoperative fasting levels) and what kind of helpers (ie, soft toy or figure) to escort the game’s character and selection of rewards they would like the game to offer.

#### Poster Walk

Next, the group was divided into pairs, and each pair was invited to take part in a poster walk with the workshop facilitator. The aim of the children’s poster walk was to inform the game design through conversation and simultaneously provide a sense of security by pairing each child with a friend. The children were asked to pick from a selection of posters the color palettes, characters, and surroundings they liked the most. Each child was given 9 stickers, 3 for each selection. In pairs, they explored the options with the workshop facilitator and then put a sticker on the posters they liked the most. When they had selected their favorite posters, they were asked why they had chosen that picture and the conversation guided them to why they liked or disliked certain things.

#### Drawings

While some children participated in the poster walk, the others sat at a table with paper and crayons. They were invited to draw a picture about hospitals to help them communicate and process their thoughts and give the researchers insight into their perceptions or knowledge about hospitals.

### Usability Testing

The usability of the game was tested by first observing children play the developed game and then interviewing them afterward. The aim of the usability testing was to evaluate the ease of use, attractiveness, and functionality of the game from the children’s perspective. In Iceland, the group comprised children (n=9, 56%) who had also participated in the game design workshops described in the Development of the Game section 1 year earlier. The testing took place in the children’s playschool. In Finland, children (n=7, 44%) were recruited for testing by convenience sampling [28]. The testing was conducted at the children’s homes (n=2, 13%) or, due to the COVID-19 pandemic, via teleconferencing software with parental assistance (n=5, 31%). The researchers documented the children’s age and gender, and data from each child were marked with a code letter to replace their name. Each session was audio- and video-recorded.

#### Observation

In Iceland, the usability testing was facilitated by 2 or 3 researchers. The children were divided into groups of 2 or 3, and each child was given a headphone and a tablet computer with the game ready to play. The children were asked to play the game, and while they did so, 1 researcher sat beside each child, observed what happened, and was available to help, when needed. In Finland, the children were observed by the researcher during gameplay via teleconferencing software in 5 cases.

To determine *ease of use,* the children were observed to see whether they were able to move between the levels and start and play the levels independently and without problems. Researchers also observed how the children solved problems during the gameplay, whether they were stuck or confused, and whether they were able to proceed within the game without help. *Attractiveness* was evaluated by observing each child’s engagement and signs of enjoyment and interest. *Functionality* was evaluated by observing whether the game functioned without technical difficulties, and if not, such difficulties were noted.

#### Interviews

After playing, the children were interviewed, either in groups (Iceland) or individually (Finland), by the facilitator about their gaming experience, using an interview framework that was developed during the game design process (Textbox 1).

**Interview framework for usability testing.**

- What did you think about Mina the Owl and the Land of Dreams—the music and the colors in the game?
- What was fun or dull, and easy or difficult in the game?
- What did you think about the exercises? When can you use these exercises? Can you show me how to do it?
- What do you think happens when you have to go to the hospital? What would you tell other children will happen if they have to go to the hospital?
- Do you feel that you would like to play the game all over again/continue playing?
- Have you used a tablet computer like this before? Have you played a computer game before?

### Data Analysis

To analyze the data, descriptive statistics and directed content analysis were used [29]. With this qualitative approach, meaning is interpreted from the content of data. Codes are derived from theory or relevant research findings and defined before or during data analysis, thus supporting or extending the existing theory. Coding is conducted with the predetermined codes, and data that cannot be coded are analyzed to determine whether they represent a new category [29].

Data analysis was performed by 1 researcher in each country (authors BI/KG and EL). To enforce reliability, a detailed analysis frame was created for the video recordings, which was discussed thoroughly and collaboratively by the research group, both before and during the data analysis, and the researchers verified coding with each other. The recordings from each session (workshops and usability testing) were watched/listened to and analyzed first, and then a summary of all sessions was written to describe the findings.

When preparing the analysis of data from the workshops, 2 categories were predetermined: *ideas about hospitals* (with subcategories on what happens in hospitals, people in hospitals, equipment in hospital) and *ideas about emotions* (with subcategories on each emotion explored, ie, fear, courage, excitement, relaxation, pain, and anxiety). The data, that is, the observations (video recordings), field notes, and drawings were analyzed from these 2 categories.

When analyzing data from the usability testing, the analytic framework included both evaluation of the child’s perspective (ease of use and attractiveness) and technical issues (functionality). Coding was performed by marking a plus (+) or a minus (–) for each factor, depending on whether there was or was not a problem requiring assistance or whether the child did or did not look to be engaged, interested, or enjoying during playing. The usability was evaluated by counting the frequencies, that is, how many children, of the 16 participants, managed to start each level and proceed with playing the game without problems and how many looked to be engaged, interested, or enjoying playing the game. Thus, possible scores ranged between 0 and 16, with higher scores indicating higher usability. A copy of the observation sheet can be found in Multimedia Appendix 1. For the interviews that followed the game-playing, *attitude toward the game* and *what happens in the game* were chosen beforehand as categories for coding.

### Ethical Considerations

The study was approved by the National Bioethics Committee in Iceland (VSN-19-093) and the ethics committee of the University of Turku, Finland (December 16, 2019). The researchers also acknowledge the United Nations’ Conventions on the Rights of the Child [30], which states children’s right to freedom of expression, including to seek, receive, and impart information and ideas of all kinds. In addition, this study was guided by the Children’s Design Guide, which aims to refine a new standard for the development of products and services that have ethics and children’s best interests at their core [31].

The parents in both countries received written information and signed an informed consent form before their children participated in the study. In Finland, the information was given to the children in a written but age-appropriate way, and the parents were asked to read the information to their children and discuss the study with them. Both parents and children signed the written consent form before participation in Finland; in Iceland, only the signature of a parent was required.

## Results

### Development of the Game

#### Workshops

**Figure 1:**
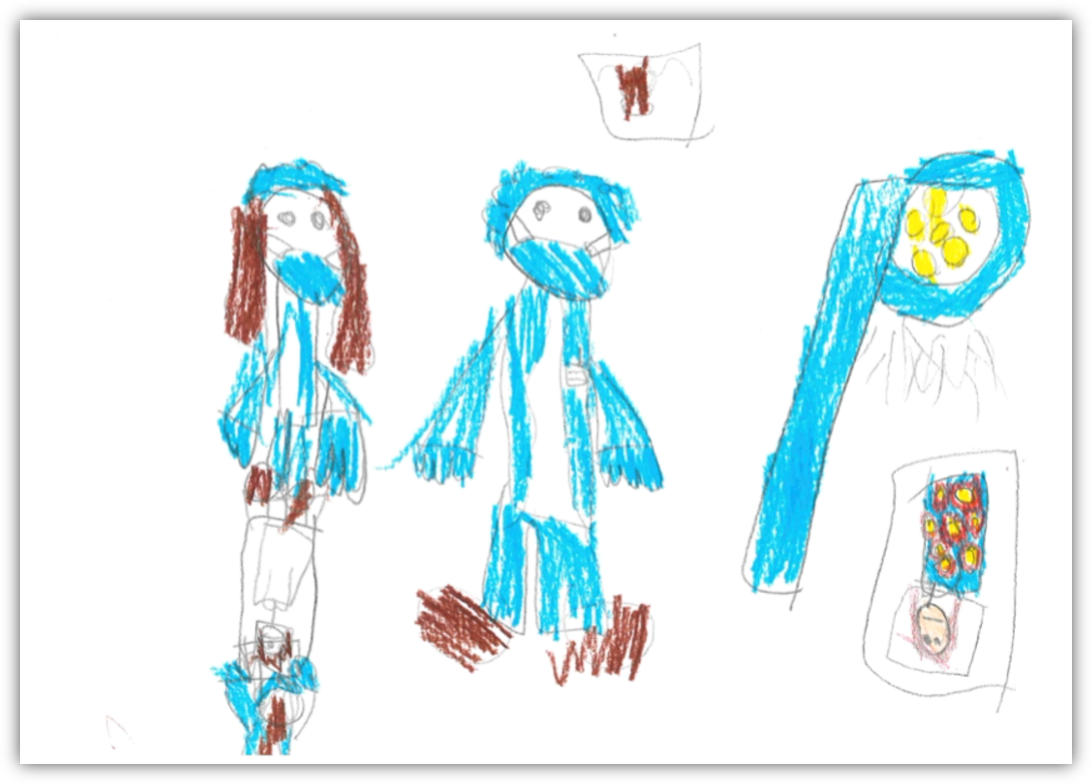
Me in a hospital (1).

**Figure 2:**
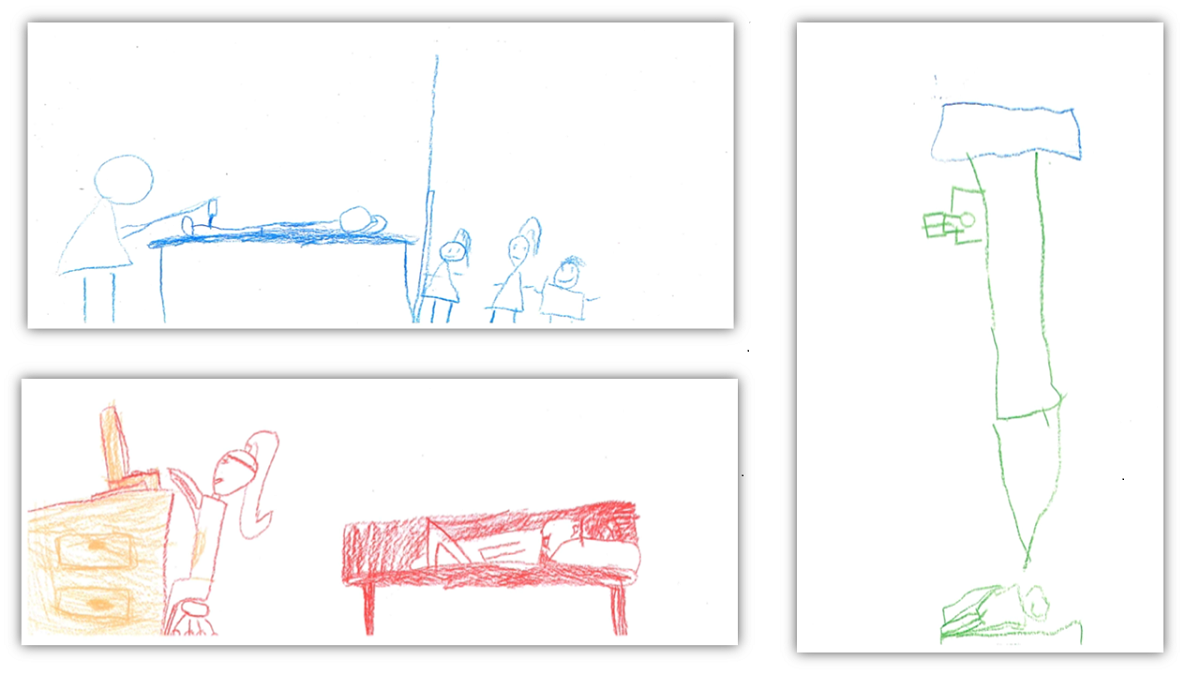
Me in a hospital (2).

**Figure 3:**
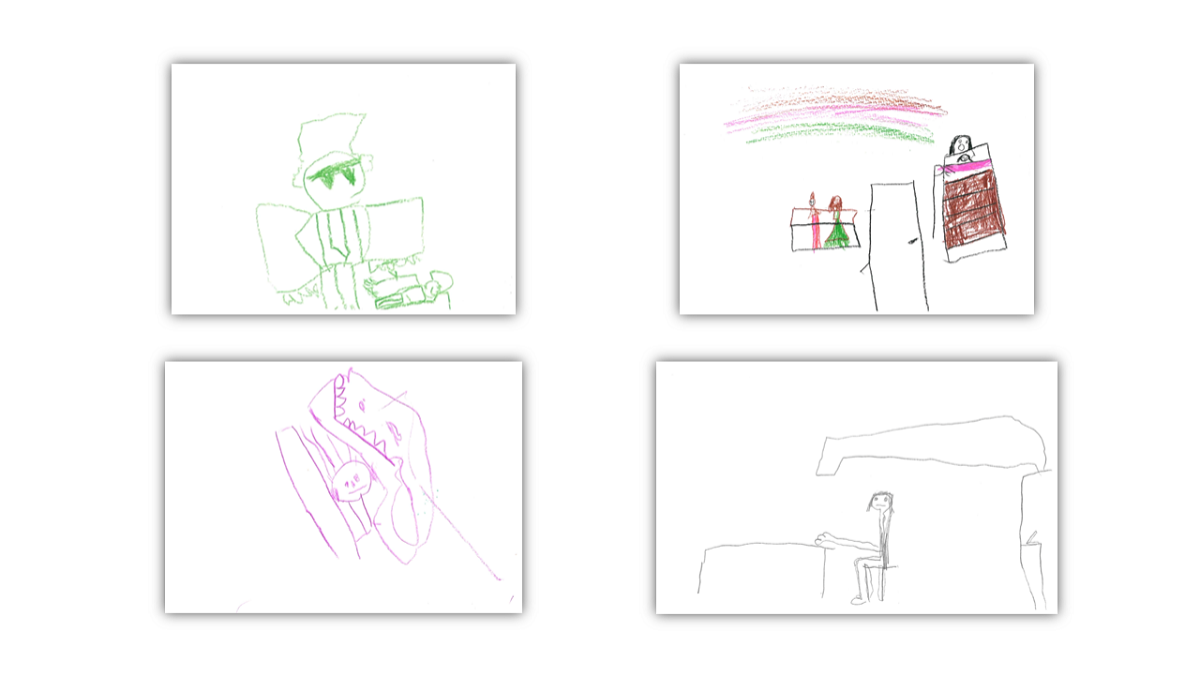
Me in a hospital (3).

A total of 26 children (13 girls and 13 boys, 50% each: Finland n=12, 46%; Iceland n=14, 54%) participated in the 1-hour-long workshops. When asked about their thoughts and perceptions concerning hospitals, the activities happening in hospitals, and the people they could expect to meet there, the children gave various responses. They perceived hospitals as places where you go “to be fixed,” “to be examined,” “they take your weight or put you to sleep,” or “they cut and there is blood.” The people they met in the hospital settings were “nurses, doctors, and patients,” and they also expected to see “ambulances” and “machines to take pictures of your bones” in the hospitals. Other hospital-related issues the children mentioned included medicines, which they referred to as something “you have to eat to get cured if you are ill.” The fun things children expected in hospitals were “prizes, toys, and juice cartons.” When children drew pictures related to their perceptions about hospitals, they illustrated themselves often as small figures against the huge hospital equipment and isolated from their loved ones. Examples of the drawings are presented in Figures 1-3.

When showed commonly used hospital equipment and asked whether they knew its purpose, the children could easily recognize the purpose of a stethoscope (“to listen to the heart”), a syringe (“for a medicine,” “for an injection, then it has a needle on”), a Band-Aid (“when there is blood,” “when it hurts”), an anesthesia mask (“you use it to breathe in medicine for your lungs”), or a medical mask (“it is this thing, when you have something, you put it on so that the bacteria won’t spread from the doctor,” “you put it on the mouth”). The drawings also represented knowledge and realistic ideas about hospital equipment, for example, the big lamp, X-ray equipment, faces with masks, and syringes (see Figures 1-3).

When discussing feelings and emotions, the children perceived *courage* as a sense or an action, for example, “you save someone,” “being alone in the dark,” “not being scared when something is exciting,” “going where there is a monster,” or “going somewhere when you don’t know what is there.” A child with hospital experience said that you need to be courageous there but will be rewarded with various prizes and treats. When talking about *pain*, the children had experienced pain in the stomach, ears, and arms and after vaccinations and pain could be relieved by medicine, massage, and rubbing; by a Band-Aid; or by going to a doctor or a hospital. Being *scared* could be related to games, such as pretending to be a ghost, scary animals (eg, lions), watching television, and nightmares, and could be fixed by going to one’s parents or other adults or cuddling a soft animal. *Relaxation* was when you are resting, doing something quietly, sleeping, or sitting. Being *excited* was both a positive experience (eg, feeling excited when going to the playground or having a birthday) and a negative one (“it is like being scared, being nervous about what is going to happen”) and could be eased by being with your parents or “just don’t go there.”

When asked what kind of “helpers” or soft toys they would like to bring with them to the hospital, the children suggested unicorns, kittens, foxes, teddy bears, and a frog. They were also asked about their favorite foods and drinks, and popular were pizza and pasta, carrots, rice pudding, chocolate milk, juice, water, hamburgers, hot dogs, and ice cream. For a reward they would like to get in the hospital, the children suggested treats, such as ice cream, a medal, a trophy, toys, and stickers.

#### Design of the Game

The design solutions were produced by using the results from the workshops, the expertise of the multiprofessional team, a literature review, and discussions with parents and clinicians. The team met regularly to exchange ideas and perspectives and find solutions that met the purpose of the game of being educational while simultaneously providing basic game elements, such as goals, fun, markers of success, and rewards. We decided to design a game that would be available in 3 languages (English, Finnish, and Icelandic), playable both in iOS and Android cell phones or tablets, and freely accessible from Google Play Store and Apple App Store. The game was designed to meet the developmental stages of 4-6-year-old children and to be played at home, with parental supervision, before the medical procedure.

The final game was programmed in the C# programming language and designed in the Unity game development environment [32]. Throughout the design process, the game was tested by children in the target group.

**Figure 4:**
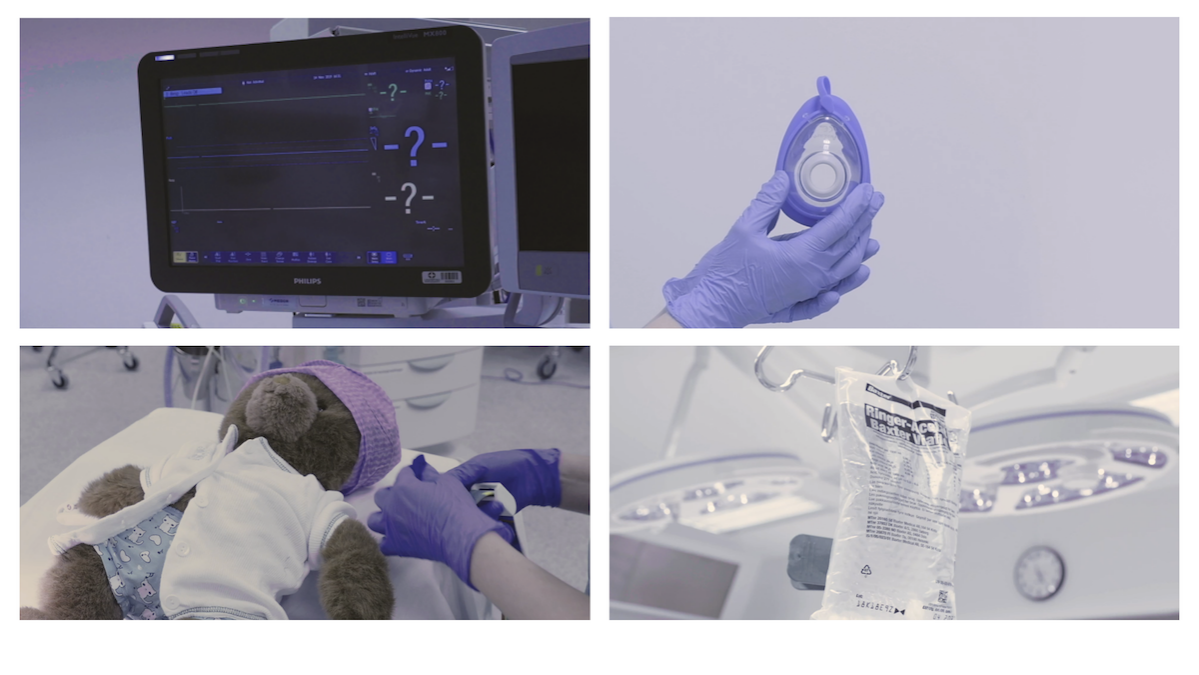
Screenshots from the hospital videos integrated in the game.

**Figure 5:**
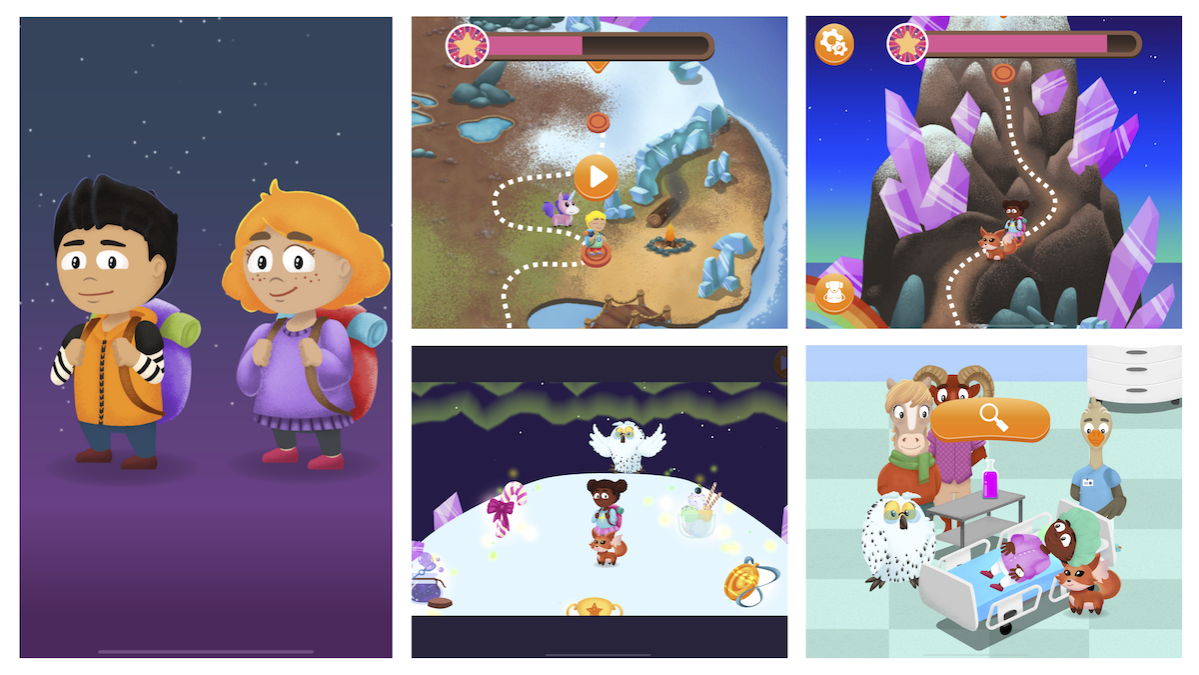
Screenshots from the different game levels.

The game is a mixture of adventurous and realistic contents and presents a child’s trip to the hospital through a landscape inspired by Icelandic nature. The child in the game is escorted by a narrator (Mina the Owl). The game starts with a short introduction and an invitation to choose from a selection of 4 avatars (characters) and 4 helpers (unicorn, fox, cat, teddy bear). The storyboard consists of 9 game levels, which are divided into 2 main sections. Levels 1-5 are set in nature, and the subject is preparation for hospitalization, that is, preoperative fasting, bathing, and teaching coping skills with positive self-talk, encouragement, and relaxation exercises. The second section, levels 6-8, introduces the hospital environment with a mixture of illustrated animations and 6 short (circa 30-second) real-life videos with a teddy bear in the role of a patient. The videos were recorded in a hospital and focus on the equipment, surroundings, and sounds that the child can expect to see and hear there. Other characters appearing are animals (horse, goose) that represent family and hospital staff. The interface includes a courage meter, which monitors the child’s success and gradually fills up during the game; pause and start buttons; and collectible items, which are retrieved during the game. Figures 4 and 5 present screenshots from the game’s interface.

The soundtrack for the game is original, and the composer’s aim was to create an atmosphere that matched the intention of the game, suggesting both mysterious adventures and a safe environment. A flute, a cello, and a vibraphone were used for the music, which was recorded in an old church in rural Iceland and then edited to match with environmental sounds in the game, both in nature and within the hospital. Table 2 presents the game’s storyboard and the tasks tested in the usability study, with information about the children’s input into the original game design.

A short trailer of the game can be viewed on YouTube (Multimedia Appendix 2).

**Table 2:** Description of the interface, related tasks, and the children’s contribution to the game design.

Interface description	Tasks for the children to engage in	Children’s input into the game design
Mina the Owl greets the child and explains the game.	Listen to the introduction.	NA^a^
Four avatars (characters) to choose from.	Choose an avatar.	NA
Four helpers to choose from: unicorn, fox, bear, and cat.	Choose a helper.	They gave a list of their favorite soft toys.
Landscape with food distributed on the ground. A stomach fills up with each collected food item.	Level 1: Collect food.	They gave a list of their favorite foods.
Mina teaches the child an exercise.	Level 2: Learn deep-breathing exercises. Choose between proceeding or replaying the level.	They showed how to blow soap bubbles and do deep-breathing exercises.
Landscape with water (river, waterfall, rain, ponds) and drinks to collect. A bottle fills up and needs to be shaken to complete the level.	Level 3: Collect drinks, shake the bottle, and drink.	They gave a list of their favorite drinks.
A rag doll and a robot appear, and Mina teaches the child to relax and repeat the movements.	Level 4: Learn relaxation. Choose between proceeding or replaying the level.	They discussed the concept of excitement and relaxation.
Landscape with bathing gear distributed on the ground.	Level 5: Collect bathing gear.	They discussed what is needed when one takes a bath.
Hospital environment with additional characters (representing hospital staff and family).	Level 6: Visit 3 rooms and find and watch 6 short videos.	They expressed through drawings and discussions that the environment can be overwhelming, with huge and bright lamps, staff with green/blue hats, parents who are isolated from you, painful procedures, etc. These findings led to the decision to add to the game both figures representing family members and short, realistic hospital-based videos.
Hospital environment, preparation for anesthesia. The child falls asleep, flies in the sky in the Land of Dreams, and then wakes up.	Level 7: Preparation room, operation room, and back to preparation room. Find and attach an intravenous infusion and a mask.	See the “Children’s input into the game design” for level 6.
Trophy room with a selection of trophies.	Level 8: Choose a trophy.	They provided ideas about attractive trophies or rewards that they would like to receive in a hospital.

^a^NA: not applicable.

#### Usability of the Game

**Figure 6:**
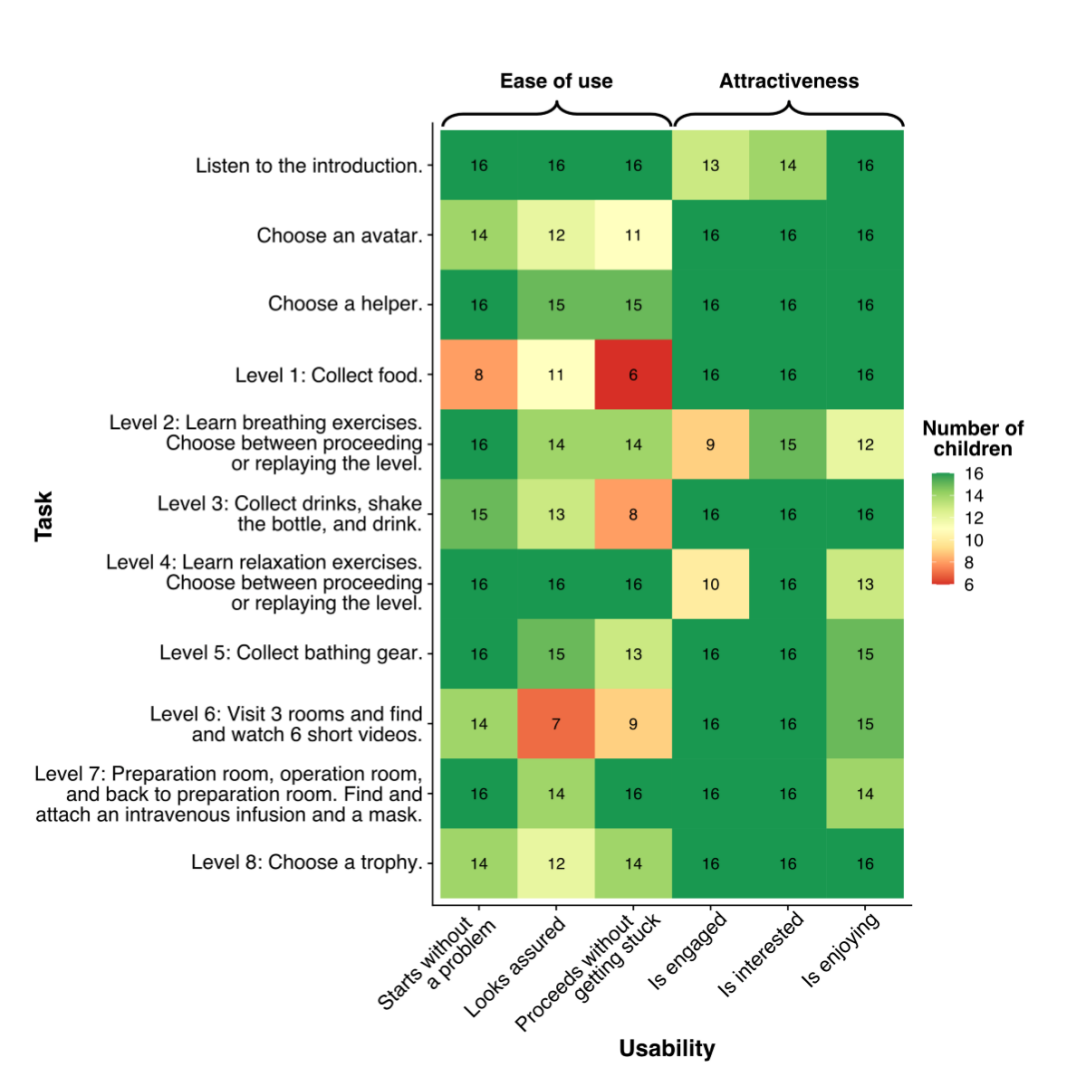
The children’s (n=16) perspective of the usability of the game.

A total of 16 children (12 [75%] girls and 4 [25%] boys: Finland n=7, 44%; Iceland n=9, 56%) participated in the usability study. Their median age was 5 (range 4-6) years. Four children had not played a computer game before, but all were familiar with a tablet computer. The results from the usability testing are presented in Figure 6.

#### Ease of Use

The assessment of *ease of use*, that is, the degree of difficulty in starting or moving between the levels, revealed that only a few problems arose with these tasks (Figure 6). No child gave up or refused to continue playing. Overall, the children began, paused, and stopped at each level without assistance or with minimal assistance and were quick to learn how to proceed.

#### Attractiveness

In the beginning, the guide and narrator, Mina the Owl, greets the player and starts the game with an introduction, and throughout the game, as each level unfolds, she breaks in with explanations. It was observed that although some of the children started to listen attentively when the owl spoke, others lost interest and got impatient to proceed or even tried to continue while she was still talking (which was not possible). During the interviews with the children, this was confirmed: they liked the owl, but she “talked too much.” This was especially evident in the 2 exercise levels, which include the deep-breathing exercises and relaxation. The children observed and listened attentively while the owl explained and encouraged them to try the exercises, but few children wanted to practice the exercises; some of them became a little distracted and wanted to proceed to the next level.

The children liked the game’s characters and helpers and chose them quickly. The unicorn and cat were the most popular helpers (6 [38%] children chose each), followed by the fox (n=4, 25%), and no one chose the teddy bear. The characters in the hospital levels representing staff and parents were perceived as a bit strange but funny as they were not humans but animals.

In general, the children showed interest and were engaged throughout the game-playing. They expressed both concentration and joy through smiling and chuckling while learning to gather the collectibles (food, drink, bathing gear) and interest, curiosity, and concentration when they entered the hospital levels. At this stage, they noticed more the courage meter and the incoming points and got more excited about this. The game finishes in the trophy room, where the children can choose different prizes. At this point, some of the children had had enough and did not want to listen to the Mina the Owl completing her talk.

During the interviews, the children could recall detailed information from the game, such as that the intravenous fluid was for surviving, and could describe the equipment in the hospital room quite well. What they found most fun was choosing the characters; walking in nature while retrieving the collectibles; eating, bathing, and drinking to get energy; going to the hospital; moving the characters; and getting the trophies. They also referred to watching the video clips as enjoyable and could easily recite what happened to the teddy bear in the hospital. At the hospital, the character flies to the Land of Dreams when the anesthesia is initiated. This was a well-recalled level. The music was perceived as nice, but some children did not notice it. All but 2 (13%) children replied that they would like to play the game again.

#### Functionality

This part assessed whether the game functioned well without technical difficulties. All the children (n=16, 100%) managed to complete the game in a similar time; it took them approximately half an hour to play (median 30 minutes, range 25-37). Although no technical issues arose during the testing in Iceland, a few functionality problems were noticed in Finland, which were repaired after the usability study.

## Discussion

### Principal Results

This paper presented in detail how preschool children were involved in the development and testing of a serious health game targeted at their own age. A well-defined and structured process was followed, linking design and research to gain better evidence on this novel approach to educating young patients. An interdisciplinary approach in 2 countries was used to ensure diversity in the game design, and the robust data collection methods and appropriate sample size ensured the quality of the study. Professionals were chosen to facilitate the workshops and usability testing and to communicate with the children in a way that was appropriate for their developmental stage.

The purpose of a study like this is not to produce data to generalize from but rather to describe in detail the process of the design and the lessons learned and thus to add to the available knowledge about child-centered design. With this paper, the authors are responding to a discourse within serious game design where researchers have called for and emphasized the importance of reporting and describing more and better the design process and the end users’ involvement and conducting research parallel to the design process [33,34]. This is important for serious games to have credibility as educational tools in health care.

### Comparison With Prior Work

Few previous studies have described similar information where the participants are only preschool children. A child-centered design of games was mainly reported from projects with the participation of children around the age of 7-11 years [22]. During the design process of Mina the Owl and the Land of Dreams, the target group, children aged 4-6 years, were able and eager to articulate their thoughts and perceptions about hospitals, related emotions, and preferences regarding various relevant issues in the game design.

#### Lessons Learned About Designing With Children

A fact relevant to the game design with this age group is that children have difficulties with abstract thinking; their size and motor skills define their abilities to handle equipment such as computers and accessories, their attention span is limited, and they mainly express themselves through playing [21,24]. They are just starting to practice their literacy skills and are becoming able to read and write simple text [35]. The game was therefore designed using audio recordings of the narratives with subtitles, and tablet computers or smartphones were chosen as platforms, both of which are easy for small children to use. The motor skills that the game-playing requires were appropriate for this age group, and the children accepted the use of tablet computers easily.

Using playful workshops, including playing a board game and encouraging expressions of ideas about hospitals through drawings, was successful, probably because these methods were appropriate to the children’s developmental stage. Their cognitive level was carefully considered when abstract concepts, such as different emotions related to hospitalization, were introduced in the game, and it was recognized that at this age, children are just starting to learn to describe their feelings and emotions [22].

The participating children were able to express their preferences about, for example, food, drinks, and toys, and perceptions of hospitals and emotions through discussions and drawings during the game development. They had a common understanding about the purpose of hospitals and what happens there and could identify the various types of hospital equipment presented to them. They used their experience, creativity, and imagination to conceptualize and explain the various emotions relevant to the game. Of special interest are the concepts of fear and courage, which are at the center of the game. The children’s drawings indicated a fear of hospitals and a fear of being alone, and pain and blood were found to be part of the expected experience. These findings supported the initial intention of the research group of letting the concept of courage and coping strategies be the focus of the game and confirmed the appropriateness of having the courage meter as a central part of the interface. These findings also led to the decision to add more characters to the game that represent the health care professionals and family members in a playful way. Drawings are a strong medium for children to convey their experience and perceptions about hospitalization [36], and their use proved to be fruitful and conveyed important information for the game design.

An important issue to consider when working with young children is the power structure linking the child, the researchers, and the game designers. Children are generally expected to be led and directed by adults, but in the developmental process of a game, it is important that the children experience trust and collegiality [22]. Their self-efficacy can be supported with such participation, and it increases the success of implementation of a game.

A child-centered design acknowledges the right of children to have a say in matters that concern them [21], but including children in research entails several ethical considerations, such as awareness of the child’s vulnerability, how to gain consent, which methods to use, and how studies are executed [37]. Other authors have emphasized the need to avoid overprotection because of vulnerability and rather view children as social actors who can have a say in their participation in research, but taking this stand requires researchers to communicate effectively with children [38].

It is important and beneficial to interview children in an environment where they feel safe and comfortable [39]. In this project, a trusting relationship and a comfortable atmosphere were created and reflected in the enthusiasm and willingness of the children to share their opinions and thoughts with the researchers. Both workshops and usability testing were conducted in a familiar and safe environment for the children, and they had support from their peers or family. Other measures that the researchers applied to create trust were to deliberately put 1 researcher with a pedagogical background in the front as the main communicator and facilitator of the workshops and usability testing; to carefully introduce themselves, explain the purpose of the game, and seek permission to work with the children; and to use play to communicate with the children and listen carefully to what they had to say. In the play schools, conversations took place on the floor, where the children could freely move, handle toys, and interact with their friends as they were assisted to communicate their thoughts and ideas. All this contributed to the success of the design process and can be strongly recommended.

Overall, the design process helped understand what hospital- and anesthesia-related subjects and concepts need to be explained in the game, and how, in order to ensure they are age-appropriate for the target group.

#### Lessons Learned About the Usability of the Game

In the usability study, the game proved to be easy to use and was accepted intuitively and easily by the children. They needed minimal assistance, even those who were not so familiar with a tablet computer, and they quickly learned how to proceed in the game. Children nowadays are often familiar with the use of smartphones; a recent study in the United States found that around 60% of children are reported to begin engaging with a smartphone before the age of 5 years [40]. However, there are households that do not support children’s use of such equipment, and therefore, computer games should be regarded as an addition to current educational practices and not a replacement.

The content of the game was chosen and designed based on evidence about what creates fear in hospitalized children and on beneficial interventions to tackle children’s perioperative anxiety. Children use many coping strategies for hospital-related fears, especially ones where they play an active role and are in control [41,42], and the strategies that were chosen for the game, that is, relaxation and deep-breathing exercises, have proven to be effective [11]. Although the children attentively watched the 2 levels with exercises, they did not join in practicing them during the usability testing, even when invited to do so. This could be a sign of no interest or that the situation and environment were not optimal. Perhaps the children did not understand that when entering the levels with the exercises, different actions were required by them, such as stepping out of the game and into practicing the exercises. This indicates that in future real situations, and for them to be useful, children will need support from parents/adults to learn and practice the game’s exercises before entering the hospital. This was an important result from the usability testing and an example of a lesson learned: children do not necessarily think or act in the way adults might suggest or suppose them to. This also highlights the importance of children participating in the development process and in the matters that concern them.

The attractiveness of the game was confirmed with the long attention span the children showed during the 30 minutes it took for them to play. They were engaged and attentive, especially enjoying the interactive parts. This time far exceeded the research group’s initial plan of the game’s length, recognizing that attentiveness in children of this age group is limited [22], but this time was needed to convey the educational content to the children. It is likely that the engagement was a result of the interactivity in the game and reward system as well as how it features both adventurous animation of nature and a realistic environment of the hospital presented with videos and addressing different actions and educational modules.

The usability testing revealed that the narrative (or the script) of Mina the Owl, who is educating and explaining, was too long, as many children became impatient waiting for the owl to finish talking so that they could proceed in the game. This led to the script being cut down when the game was revised after the testing and before the game was published.

### Limitations

The lack of a validated tool to use in the usability study can be regarded as a limitation of this study. The analysis of each of the video recordings was performed by 1 researcher, but the researchers created a detailed analysis plan together beforehand and followed it rigorously. Furthermore, it may also be regarded as a limitation that it was not possible to ensure that an equal number of boys and girls participated in the usability study, which may have affected the results.

The study has provided important information, both about designing a game with young children and also about this particular game, and the findings led to adjustments and changes before the game was published. The study also provided further ideas about how the game could be improved in its next versions. A future feasibility and implementation study within the clinical environment will explore and provide evidence on the efficacy and effects of the game on children’s perioperative anxiety, self-efficacy, knowledge about what to expect in the hospital/clinic, and coping strategies when undergoing anesthesia.

### Conclusion

Preschool children can actively participate in developing educational, computer-based games as a target group and can provide important information about their preferences, understanding of abstract concepts, and the usability of a game. It is important to use child-oriented methods, such as playful workshops, in the design process that safeguard children’s rights in research, support trust in the researchers, and are conducted in a safe environment. The usability of the tested game was high in terms of ease of use, attractiveness, and functionality, but long narrative parts were rather challenging for this age group. Important lessons were learned by the study to take into the next phase, where the effectiveness, efficacy, and feasibility of implementing the game in practice will be explored.

## Appendix

Multimedia Appendix 1 The structured observation schedule used in the usability testing.

Multimedia Appendix 2 Trailer of the game.
