# Supplementary figures and images for: Developing a Health Game to Prepare Preschool Children for Anesthesia: Formative Study Using a Child-Centered Approach
Source: JMIR Serious Games. 2022 Jan 20;10(1):e31471. doi: 10.2196/31471 (PMC8814931; doi:10.2196/31471)

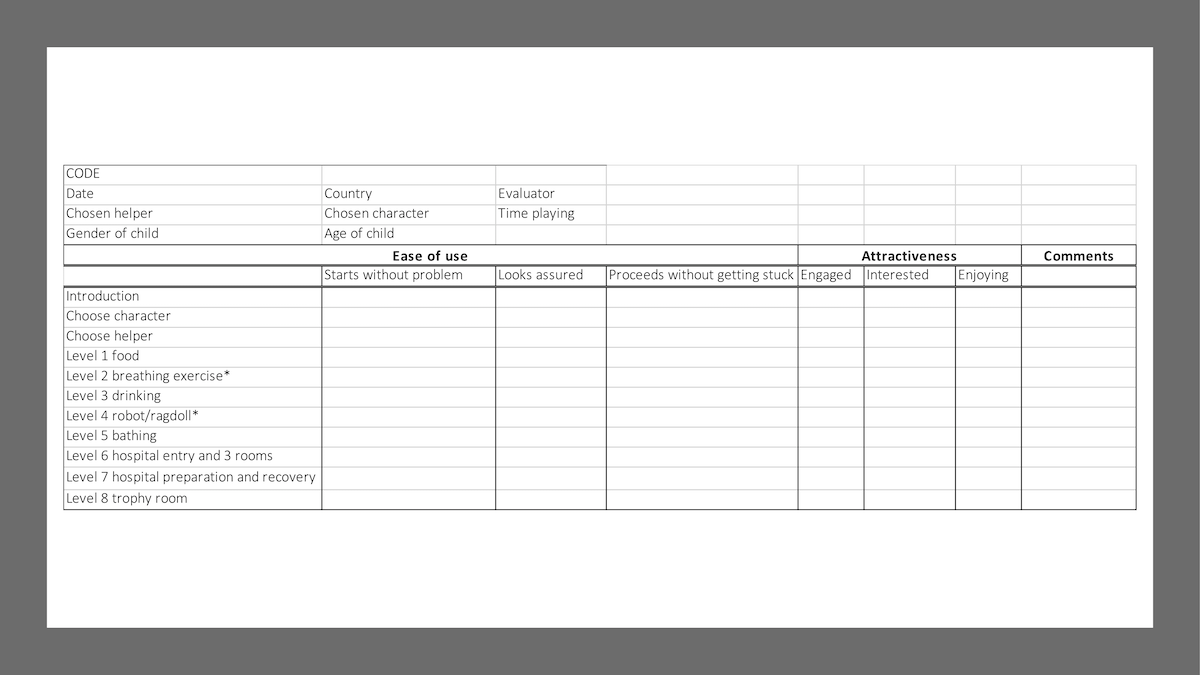

Supplement: Multimedia Appendix 1 [file games_v10i1e31471_app1.png]
